# Implementing a fully computed tomography-free online adaptive palliative radiotherapy: a one-visit workflow^☆^

**DOI:** 10.1016/j.phro.2025.100896

**Published:** 2025-12-20

**Authors:** Ashaya T. Jaglal, Koen J. Nelissen, Angelique R.W. van Vlaenderen, Amy L. de la Fuente, Famke L. Schneiders, Peter S.N. van Rossum, Jan Wiersma, Wilko F.A.R. Verbakel, Suresh Senan, Jorrit Visser, Eva Versteijne

**Affiliations:** a Amsterdam UMC Location Vrije Universiteit Amsterdam, Department of Radiation Oncology, Amsterdam, the Netherlands; b Cancer Center Amsterdam, Cancer Treatment and Quality of Life, Amsterdam, the Netherlands; c Varian Medical System, Inc., a Siemens Healthineers Company, Palo Alto, USA

**Keywords:** Online adaptive radiotherapy, Cone beam computed tomography, Palliative care

## Abstract

**Background and purpose:**

Same-day palliative radiotherapy requires rapid workflows, but conventional computed tomography (CT)-based workflows cause delays and strain resources. Advances in cone-beam CT (CBCT) enabled accurate dose calculation and planning without a planning CT. This study evaluated the feasibility and efficiency of a fully CT-free online adaptive workflow for same-day palliative radiotherapy using high-quality CBCT.

**Methods and materials:**

This prospective study enrolled sixteen patients between January–May 2025, of whom fifteen completed same-day treatment. Eligible patients were referred for single-fraction palliative radiotherapy (8  Gy) to non-mobile target volumes. No planning CT was acquired; instead, a reference plan was generated on a phantom with standardized beam setups and planning objectives. On the treatment day, planning and delivery were performed on the Varian Ethos 2.0 platform using HyperSight CBCT, providing more accurate Hounsfield Unit imaging for automated organs at risk segmentation and target definition. Plans were adapted online and delivered while patients were on the couch. Workflow times, plan quality, and patient characteristics were studied.

**Results:**

All fifteen treatments were delivered successfully. All plans met clinical objectives, with planning target volume coverage exceeding required thresholds. The CT-free workflow reduced median departmental time to 73 min, including 28 min in the treatment room, compared with 335 min in a conventional CT-based workflow. In one urgent case, referral-to-treatment time was 2.5 h. Repeated CBCTs were required in 7 patients.

**Conclusions:**

A fully CT-free workflow for palliative radiotherapy is feasible and efficient, enabling same-day treatment, reduces departmental workload, and is well-suited for urgent cases requiring rapid intervention.

## Highlights

• A direct-to-treatment workflow enabled same-day palliative radiotherapy.
• Same-day treatment was completed in 15 patients in this feasibility study.
• Median departmental time was 73 min, including 28 min in-room.
• All treatment plans achieved over 99 percent target coverage.

## Graphical abstract

**Figure:**
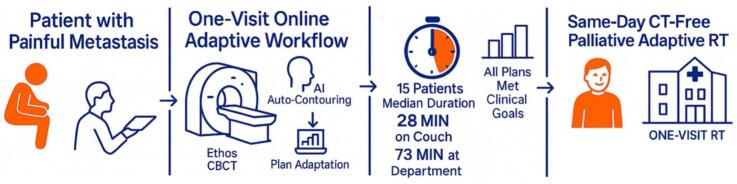

## 1 Introduction

Palliative radiotherapy (RT) for patients with symptomatic bone metastases reduces pain, preserves function and improves quality of life. A single fraction of 8 Gy is recommended for pain control [1,2]. The RT workflow for palliative indications often takes several days, from first consultation with a radiation oncologist (RO) to treatment. A planning CT (pCT) is acquired in the treatment position, typically on a flat tabletop with positioning aids to ensure reproducible position. A CT number-to-density table for the CT scanner allows for precise dose calculation. These steps introduce delays and require patients to either return on a separate day for treatment or wait in the department. To streamline this workflow, studies using available diagnostic CTs (dCTs) for both target contouring and treatment planning have concluded that dCTs can serve as a time-efficient alternatives to pCTs in palliative treatment, without compromising quality [[3], [4], [5], [6], [7]].

Introduction of so-called online adaptive radiotherapy (oART) allows for treatment plans to be adapted to the day’s anatomy and positioning, capabilities which were previously unavailable for image-guided radiotherapy (IGRT). This approach improves dose precision, thereby minimizing radiation to organs at risk (OARs) and potentially allowing for dose-escalation to tumors [8]. Recently, the FAST-METS study demonstrated that an oART approach using dCTs for planning shortens treatment workflows [9,10].

Several challenges remain when using dCT-based workflows. A suitable recent dCT may be unavailable in emergency situations and when patients are transferred from other hospitals. Occasionally, only MRI scans may have been performed or available. Furthermore, dCT-based workflows involve multiple preparatory steps, involving radiotherapy technicians (RTTs) for preparing contours and treatment planning. The current shortage of RTTs [11] favors reducing RTT involvement for treatment preparation, and the workflow can also lead to faster palliative radiotherapy for emergencies including metastatic spinal cord compression, where prompt intervention can significantly improve patient outcomes, and prevent irreversible damage [12,13].

We developed a fully CT-free oART workflow enabling a direct-to-treatment approach within a single hospital visit. This workflow requires no pCT or simulation, with all pre-treatment steps, including dose calculation and plan generation, conducted online using cone-beam CT (CBCT). Advances in CBCT software and hardware provide Hounsfield Units (HU) accuracy comparable to conventional CTs [[14], [15], [16]], which allows for pre-planning steps to be performed without a prior CT. Here, we describe the first clinical experience using this CT-free workflow for palliation using limited RTT resources, minimizing time at the department, and improving patient comfort.

## 2 Methods and materials

This prospective single-arm, fully CT-free cohort study (FAST-METS 2.0) was conducted at the Amsterdam University Medical Center, the Netherlands. Ethical approval for use of anonymized clinical data was obtained from the institutional review board in March 2025 (IRB 2025.0173). The study was deemed not to fall under the Dutch Medical Research Involving Human Subjects Act (non-WMO).

### 2.1 Patient inclusion

Patients were eligible if referred for palliative radiotherapy to non-mobile target volumes, after case review by two ROs. Additional criteria included available diagnostic imaging no older than six weeks. All patients received a single-fraction dose of 8 Gy. Informed consent for data analysis was obtained during the patients clinic visit.

### 2.2 CT-free treatment workflow

The RO conducted the first consultation for eligible patients by telephone to confirm the correlation of pain symptoms with findings observed on diagnostic imaging, before patients were scheduled for the CT-free workflows.

To standardize planning, a general planning template was developed and applied across all target sites (Supplementary materials A, Table S1). The template included planning objectives for relevant target volumes, gross tumor volume (GTV), clinical target volume (CTV), and planning target volume (PTV), as well as OARs. This template facilitated automated on-couch contouring and dose optimization, by incorporating all potentially relevant OARs. For plan optimization, only OARs located in the proximity of PTV, namely 10 cm in the transverse plane and 2 cm in the cranial-caudal direction, were assigned specific dose constraints. PTV margins of 5–8 mm were selected by the RO, depending on individual patient characteristics.

**Fig. 1:**
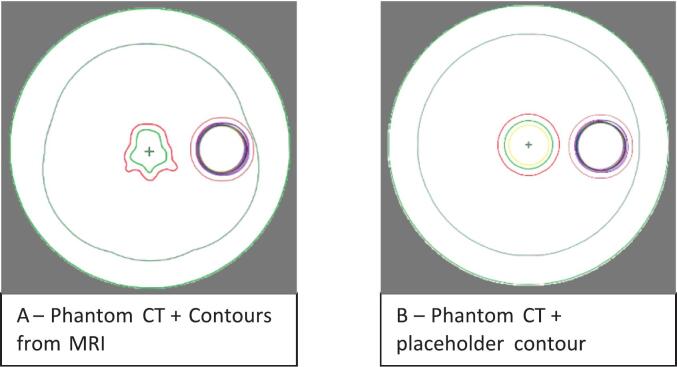
Phantoms in transversal plane. A: with propagated clinical target volume from MRI, B: with placeholder target contours. CTV in green; planning target volume in red; organs at risk placeholders in the other colors. (For interpretation of the references to colour in this figure legend, the reader is referred to the web version of this article.)

For the oART workflow, the treatment planning system (Ethos 2.0; Varian Medical System, inc., a Siemens Healthineers Company, Palo Alto, USA) requires creation of a reference plan during the pre-treatment phase. For generating on-couch adaptive treatment plans, identical beam setups and planning objectives, as for the reference plan, were used. To meet this software requirement, a CT image of a cylindrical phantom was used to substitute for an actual planning CT. The phantom measured 40 cm in diameter and 80 cm in length, with a uniform HU value of 0. A structure set comprising placeholder target volumes and OARs was generated on the phantom CT, based on the predefined planning template. Two workflows were implemented using this phantom (Fig. 1). In workflow A, the CTV was manually contoured on an MRI and subsequently propagated to the phantom. In workflow B, the phantom’s initial placeholders were used for all target volumes. These workflows allowed for standardized setup when generating reference plans and ensured compatibility with the system's automated contouring.

The beam configurations used in both the reference and adaptive plans were standardized intensity-modulated radiotherapy (IMRT) setups using a 6 MV FFF beam. Setups consisted of either a 9-field equidistant beam arrangement, or a 7-field lateral beam arrangement for laterally located tumors (Supplementary materials A, Fig. S1).

Treatments were scheduled in 30 min time slots. On treatment day, an RO consultation and clinical examination verified the target site, explained the treatment procedure and scheduled treatment directly thereafter. The RO communicated target locations to RTTs, who positioned the patient in the treatment room to align the target approximately at the isocenter of the linear accelerator (LINAC). Setup was performed using either a deflated vacuum mattress or a 5 cm foam mattress to ensure stability and patient’s comfort. Treatments were delivered using an Ethos LINAC equipped with a HyperSight CBCT (HS-CBCT) imaging panel (Varian). Treatment HS-CBCT images were acquired using Acuros iterative reconstruction to ensure accurate HU and optimal image quality [17]. Previous studies have validated the HS-CBCT HU accuracy for dose calculation [18,19], demonstrating the suitability of recent CBCT technology for clinical dose calculations. Following HS-CBCT acquisition, review of anatomy step was performed after body and OARs were automatically delineated using AI-based segmentation. As recommended [20], all AI-generated contours were visually reviewed by RTTs to ensure anatomical plausibility before use. No manual adjustments were permitted for this procedure to ensure speed. An offline review of the generated OAR contours was performed (Supplementary materials B).

During target review, the workflows diverged as follows. In workflow A, a CTV originally contoured on diagnostic MRI, was propagated to the CBCT and positioned using translations and rotations. In workflow B, placeholder target contours were removed, and the CTV was manually contoured by the RO while patients remained on the treatment couch. After acceptance of target contours, the PTV and other structures derived from CTV delineation or AI-delineated OARs were automatically generated. Based on these contours, a treatment plan was optimized. Plans were reviewed by both RO and RTTs before delivery, including inspection of the 95 %, 50 % and 107 % isodose distributions in order to ensure target coverage and dose conformity. A secondary plan check was performed using Mobius3D (Varian). Immediately prior to treatment delivery, a CBCT was acquired for final position verification.

### 2.3 Data collection and analysis

**Fig. 2:**
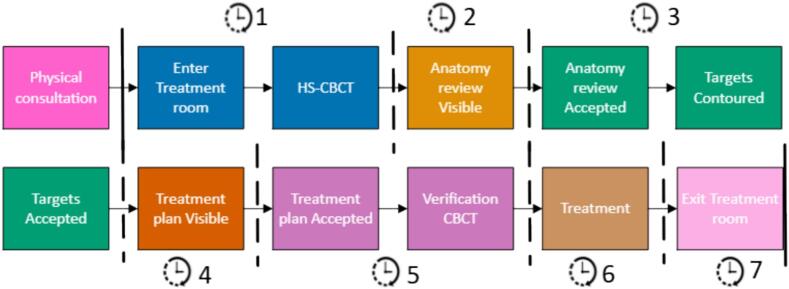
Simulation-free CT-free palliative radiotherapy workflow. The full procedure is divided into seven different timeframes (TF1–TF7), covering each key step from consultation to treatment delivery and patient exit. CBCT: cone-beam CT. HS: HyperSight.

To evaluate workflow efficiency, time at the start or end of several phases during the workflow were recorded either manually by RTTs or extracted from the DICOM data. This allowed calculation of time intervals, including time from referral to treatment, and specific timeframes on the day of treatment. The latter were categorized into seven distinct timeframes (TF1–TF7), corresponding to the stages illustrated in Fig. 2. The first timeframe (TF1) extended from entry into treatment room, patient setup until the end of the HS-CBCT used for treatment. TF2 encompassed AI-based contouring of the body and OARs. During TF3, either the CTV was propagated to its correct location (workflow A), or placeholder target structures were removed, and the CTV was manually delineated on the CBCT (workflow B). TF4 encompassed plan generation and optimization. Once the treatment plan was visible, TF5 began where the plan was checked, approved, and ended after the second CBCT was made for position verification. TF6 corresponded to the actual treatment delivery, and finally TF7 covered the time from completion of treatment to the patient's exit from the treatment room. These times were used to assess the efficiency of the FAST-METS 2.0 workflow.

Patient demographics and clinical data were collected, including age, Karnofsky Performance Scale before treatment and tumor characteristics. Treatment plan parameters analyzed included PTV V_95%,_ CTV V_95%_, and the number of monitor units (MU) delivered. Post-treatment, all data was exported from the Ethos system and anonymized for further analysis. Time points, clinical objectives, and patient characteristics were stored in a Castor Electronic Data Capture database (version 2025). Detailed treatment plan outcomes are reported in Supplementary materials C. Data were compared with the previously published FAST-METS cohort [9] (Supplementary Materials D), confirming comparability between populations.

### 2.4 Patient flow and imaging details

Between January-May 2025, sixteen patients were enrolled in this study but one failed to complete treatment. In the case of the latter, the HS-CBCT was used to plan an IGRT-based treatment for delivery on the following day. All fifteen patients succesfully completed same-day CT-free RT. The CTV of one patient was contoured on MRI and propagated to the CBCT using the phantom (workflow A). In all other patients (n = 14) CTV contouring was performed on on-couch CBCT (workflow B). Diagnostic imaging reviewed on a separate screen at treatment console included a CT (n = 7 patients), MRI (n = 4), and PET-CT (n = 4). The median interval from diagnostic imaging to treatment was 13 days (range: 0–41 days). Median time from referral to treatment was 5 days (range: 2.5 h–9 days). Patient characteristics are summarized in Table 1.

**Table 1:** Patient characteristics.

**Median age (in years) [range]**	72 [50–86]
**Gender**	**Number (percentage)**
Men	8 (53)
Women	7 (47)
**Treatment site**	**N (%)**
Lumbar spine	4 (27)
Thoracic spine	6 (40)
Pelvis	4 (27)
Scapula	1 (7)
**Primary site**	**N (%)**
Prostate	5 (33)
Melanoma	2 (13)
Kidney	2 (13)
Multiple myeloma	2 (13)
Breast	1 (7)
Esophagus	1 (7)
Colon	1 (7)
Lung	1 (7)
**Karnofsky Performance Score before treatment**	**N (%)**
50	1 (7)
60	2 (13)
70	6 (40)
80	6 (40)

## 3 Results

All fifteen treatment plans met the predefined clinical goals. Dose coverage exceeded the required thresholds, with all PTV V_95%_ > 99.1 % and CTV V_95%_ > 99.6 %.

### 3.1 Timeframes

The median time spent in the department from consultation to exit from the treatment room was 73 min (range: 56–115 min), of which 28 min (range: 22–55 min) was representative of the time inside the treatment room, see Table 2.

**Table 2:** Median and range of different treatment timeframes in the treatment room. CBCT: cone-beam CT, AORs: Organs at risk, CTV: clinical target volume.

**Treatment stage**	**Median [range], minute:second**
TF1 (patient set up − treatment CBCT)	7:55 [5:06–31:08]
TF2 (AI contouring body/OARs)	1:43 [1:35–1:57]
TF3 (adaptation/contouring CTV)	5:32 [3:24–15:30]
TF4 (plan generation)	1:54 [1:38–2:31]
TF5 (plan review and second CBCT)	3:17 [2:44–4:05]
TF6 (treatment delivery)	5:42 [2:48–7:42]
TF7 (patient exiting the treatment room)	1:14 [0:10–2:26]

**Fig. 3:**
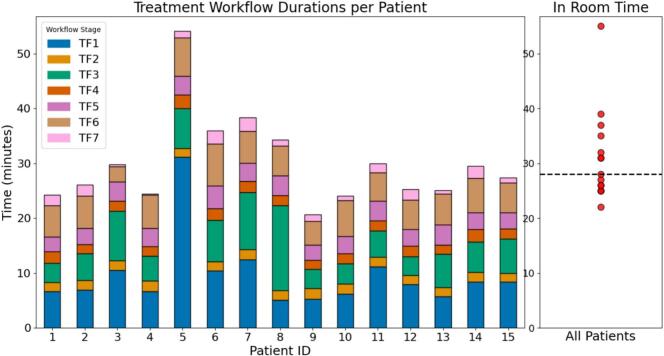
Stacked bar chart showing treatment workflow in timeframes (TF1–TF7) per patient (n = 15): TF1: patient setup and treatment CBCT; TF2 and TF3: AI-based and manual contouring; TF4: plan generation; TF5: plan review, approval, and second CBCT; TF6: treatment delivery; and TF7: patient exit. The adjacent scatterplot summarizes the total in-room time across all patients with the dashed line indicating the median.

**Fig. 4:**
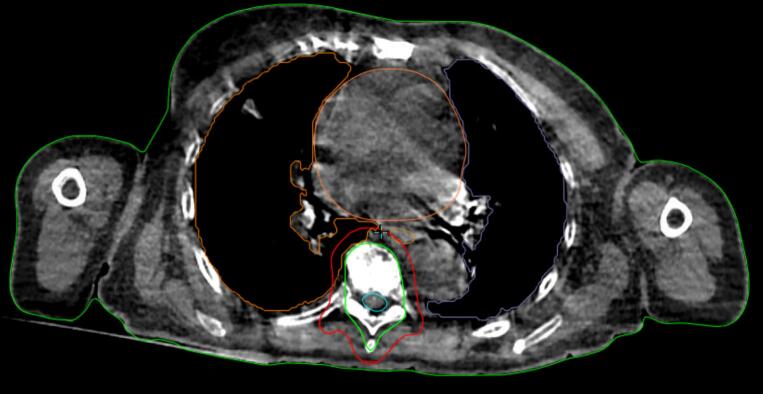
HyperSight cone-beam CT with AI-based organs at risk contouring and manual on couch clinical target volume (green) contouring used for treatment planning for patient 7. Planning target volume in red. (For interpretation of the references to colour in this figure legend, the reader is referred to the web version of this article.)

Individual workflow durations for all patients are shown in Fig. 3. Patient 1 followed workflow A. Patient 7 was treated 2.5 h after first referral and the CBCT used for planning, with AI-based OAR contouring and manual on-couch CTV contouring, is shown in Fig. 4. In patient 5, beam configuration and PTV margins were adjusted, resulting in changes to reference plan and additional CBCT acquisitions, reflected in a longer TF1 duration.

Repositioning and repeat CBCT acquisitions were necessary in patients 2, 3, 6, 7, 11, and 12, each requiring two CBCTs, while Patient 15 required three. Patient 8 had a longer contouring time (TF 3) compared to the median. From patient 10 onwards, a medical physics expert (MPE) was not routinely present during the procedure. From patient 12 onwards, a 5 cm foam mattress was used instead of a vacuum mattress.

## 4 Discussion

This study demonstrated the feasibility of delivering fully CT-free treatments in 15 cases, with a median in-room time of 28 min. Two-thirds of patients spent ≤1.5 h in the department. In one urgent case, the workflow enabled a referral to treatment within 2.5 h. Treatment goals were achieved in all cases by using a general planning template and predefined beam configurations, supporting the robustness of our workflows across anatomical sites. This template-based workflow eliminates the need for individualized pre-planning, thereby reducing RTT workload and enabling faster radiotherapy.

While oART is generally more time consuming than conventional IGRT or offline ART[8], treatment times in this study were comparable to our original FAST-METS trial, which reported a median in-room time of 27 min [9,10]. This was notable given additional time requirements for manual on-couch contouring by ROs in this study. The treatment room entry and exit times were recorded manually by RTTs and may therefore be subject to minor timing inaccuracies. However, the overall pre-treatment process was shorter as individualized treatment planning was not necessary. Importing the phantom and applying the general template and beam setup in the planning system would be possible within 15 min, a step that could be omitted totally if the Ethos platform were to permit treatment initiation without a prior reference plan. Eliminating the requirement for a reference plan would further reduce preparation time for RTTs and limit use of departmental resources. For comparison, our regular palliative workflow involves a 30 min CT time slot and a 20 min treatment session. Furthermore, omitting a CT will save approximately 20 min of RTT time and free-up CT scanner slots.

While the Ethos system represents a higher investment than a CT simulator, the on-couch time with this procedure remains comparable. Of note, our routine emergency “one-stop-shop” workflow, which includes same-day consultation, pCT, contouring, treatment planning, and radiotherapy, entails a median time at the department of 335 min per patient [10], compared to just 73 min with the workflow used in this study. Reductions in departmental time directly benefit patients by reducing their discomfort and fatigue, which is a meaningful consideration for palliation in an outpatient setting.

The use of HS-CBCT eliminated the need for synthetic CTs and deformable image registration from non-treatment dCTs. This reduces uncertainties and allows reliable use of equidistant IMRT beam setups. Use of synthetic CTs were less dependable due to inconsistent arm and diaphragm positioning. Expanding the number of fields compared to the original FAST-METS protocol [10] further enhanced dose conformity and improved sparing of OARs [21]. This is advantageous in palliative settings where minimizing toxicity with concurrent systemic therapies is desirable [22]. Recent studies have validated the dosimetric accuracy and planning reliability of HS-CBCT systems [[14], [15], [16],^18^]. Additionally, the greater flexibility in patient positioning unconstrained by prior simulation setup, allows patients to lie in comfortable positions. The observed consistency in applying a standardized 9-field or 7-field lateral beam configuration across diverse anatomical locations, including the spine, pelvis, and scapula, underscores the robustness and generalizability of the template-based approach. Moreover, the successful use of a CBCT-based contouring workflow in 14 of 15 patients suggests that MRI- or CT-based pre-contouring is not routinely necessary in most cases. Two workflows were implemented; workflow A was used in only one patient but may be preferable for large target volumes because it allows pre-contouring on diagnostic imaging, thereby reducing on-couch contouring time. It may also be advantageous for complex soft-tissue anatomy or when MRI contrast influences target delineation.

Our findings suppored the hypothesis proposed by McGrath et al. [23], whose phantom simulation study suggested that fully CT-free adaptive radiotherapy is clinically feasible. Our study confirms that such workflows are achievable in routine patient care with minimal resource requirements. Although this study used the Ethos platform with HS-CBCT, the concept is not platform dependent as any oART workflow with HU-accurate CBCT and adaptive planning tools could achieve the same approach.

During initial implementation, additional CBCTs were acquired in several patients due to suboptimal positioning relative to the imaging isocenter, resulting in incomplete imaging of the target or insufficient space for the PTV margin. This prolonged TF1 in individual cases. Patient 8 had a longer TF3 due to additional OAR delineation to spare adjacent bone. However, such changes are not unexpected in this patient population and illustrate the benefits of oART, which enables on-couch adaptations, providing the needed flexibility. Despite a requirement for the RO to be available for on-couch contouring, this workflow proved operationally feasible. Prior to implementation, an end-to-end test was performed to validate the CT-free workflow After the first ten treatments had been performed, the presence of an MPE at the unit was no longer considered necessary as safety had already been established through testing, RO/RTT review, and Mobius. As RTTs at the LINAC did not have direct access to the diagnostic imaging within the system, this could complicate precise setup. This highlighted the importance of clear communication between ROs and RTTs to reduce in-room times.

Several practical considerations and limitations should be noted. Introducing a foam mattress may improve patient comfort and setup reproducibility. Furthermore, the workflow implementation relied on the availability of recent diagnostic imaging. While HS-CBCT for on-couch contouring and dose calculation mitigates the clinical impact of anatomical differences between the prior diagnostic scan and the CBCT, clinicians must be aware of potential progression or target volume changes.

The dCT images available for some patients were intentionally excluded from oART planning in order to evaluate the workflow within standard working hours and to minimize departmental resources. CT and PET-CT generally aid anatomical landmark identification on CBCT more than MRI, which can prolong on-couch CTV delineation. Finally, the generalizability of this workflow to other institutions requires further validation.

Future research should explore the application of the CT-free adaptive workflow in broader clinical scenarios. One key area is its use in multi-fraction treatment regimens which could expedite treatment initiation in other patient categories. Further dose escalation within this workflow could be explored as higher radiation doses have been associated with improved pain relief and local control in metastatic settings [24]. We believe that the adaptive capabilities of this workflow may facilitate safe, faster and effective delivery of escalated doses.

The fully CT-free adaptive FAST-METS 2.0 workflow enables efficient delivery of palliative radiotherapy in a single hospital visit, minimizes delays in treatment initiation, and reduces the use of RTT resources. Standard templates and beam setups enable its use across different anatomical sites. These findings supported broader implementation of this approach, particularly in emergency settings, where rapid access to treatment and minimal resource requirements are critical.

## Declaration of competing interest

The authors declare the following financial interests/personal relationships which may be considered as potential competing interests: Wilko Verbakel is employed by Varian, a Siemens Healthineers Company.

The department received a research grant from Varian, a Siemens Healthineers Company (Palo Alto, California).
