# Supplementary material for: Implementing a fully computed tomography-free online adaptive palliative radiotherapy: a one-visit workflow
Source: Phys Imaging Radiat Oncol. 2025 Dec 20;37:100896. doi: 10.1016/j.phro.2025.100896 (PMC12800496; doi:10.1016/j.phro.2025.100896)
Supplement: Supplementary Data 1 [file mmc1.pdf]

Supplementary materials

Supplementary materials A

Table S1 Planning directives general treatment template. OARs that could be in the ring: Bowel, Kidneys, Lungs, Rectum, Uterus/Prostate, Stomach, Liver, Spleen, Heart, Parotids. PTV: Planning target volume, GTV: Gross target volume, CTV: Clinical target volume, OARs: Organs at risk

**Planning directives**	**Priority**
**PTV**	
V95%≥98%	1
D99%≥85%	1
D0.10cm^3≤110%	1
**GTV+CTV**	
V100.0%≥60% Variable: V100%≥40%	1
**Spinal canal+0.3cm**	
D0.10cm^3≤8.4Gy	2
**Esophagus+0.3cm**	
D0.10cm^3≤8.4Gy	2
**OARs within OAR-ring**	
D0.10cm^3≤8.4Gy	2
V2Gy≤30%	2
V4Gy≤15%	2
V6Gy≤5%	2

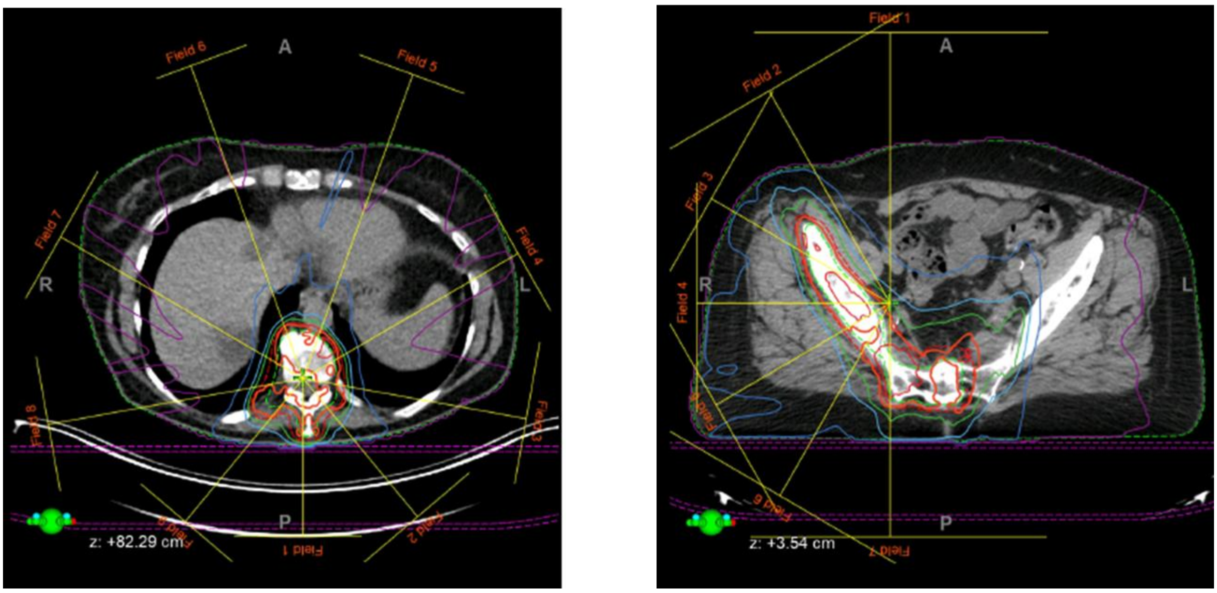

Figure S1 Beam setups 9 field equidistant and 7 field lateral

Supplementary materials B

Offline review of AI-generated contours

Images from all 15 patients underwent offline review of the AI-generated organs at risk (OAR) contoured within the OAR-ring, which was 10 cm transversely and extended 2 cm in cranial-caudal direction. In total, 65 OAR contours were reviewed across 13 different structures. Each contour was independently evaluated for accuracy ("correctly contoured") and clinical usability ("usable for optimization in palliative planning") by a medical physics expert and a researcher; cases considered to be difficult were additionally reviewed by a radiation oncologist. The results of this review are summarised in Table S1.

Overall, approximately two-thirds of all AI contours were directly usable. AI performed consistently well for large, clearly defined thoracic organs such as the lungs, liver, and heart, where all contours were rated as accurate and clinically usable. More variability was observed for smaller or anatomically complex structures, particularly the kidneys, bowel, esophagus, and stomach, which showed higher rates of suboptimal delineation. One patient presented with a diaphragmatic hernia, which may have contributed to contouring inaccuracies for upper abdominal organs due to altered organ position.

Although the AI model used does not produce perfect contours, contours were deemed sufficiently accurate for use in plan optimization for palliative care, where the previous standard approach was often simple anterior–posterior/posterior–anterior irradiation.

Table S1 Accuracy and clinical usability of auto-generated organs at risk (OAR)

Auto-generated contour	Occurrence	Correctly generated (%)	Suitable for plan optimalisation (%)
**Lungs**	7	100	100
**Esophagus**	6	50	66
**Spinal canal**	15	87	93
**Bowel**	9	44	89
**Liver**	6	100	100
**Bladder**	2	50	100
**Rectum**	1	100	100
**Prostate**	1	100	100
**Heart**	4	100	100
**Kidney Left**	4	0	25
**Kidney Right**	4	25	75
**Stomach**	3	33	66
**Spleen**	3	66	66

Supplementary materials C

Treatment plan outcomes

Table S2 Treatment plan outcomes for all patients (n=15)

**Median CTV (cm^3)**	138 (range: 47–386)
**Median PTV (cm^3)**	298 (range: 144–714)
**CTV $V_{95\%}$**	>99.6%
**PTV $V_{95\%}$**	>99.1%
**Median MU**	2982 (range: 2120 – 4033)
**Beam Configuration**	**N (%)**
9-field IMRT	13 (87)
7-field IMRT	2 (13)
**PTV margins**	**N (%)**
5 mm	3 (20)
7 mm	4 (27)
8 mm	8 (53)

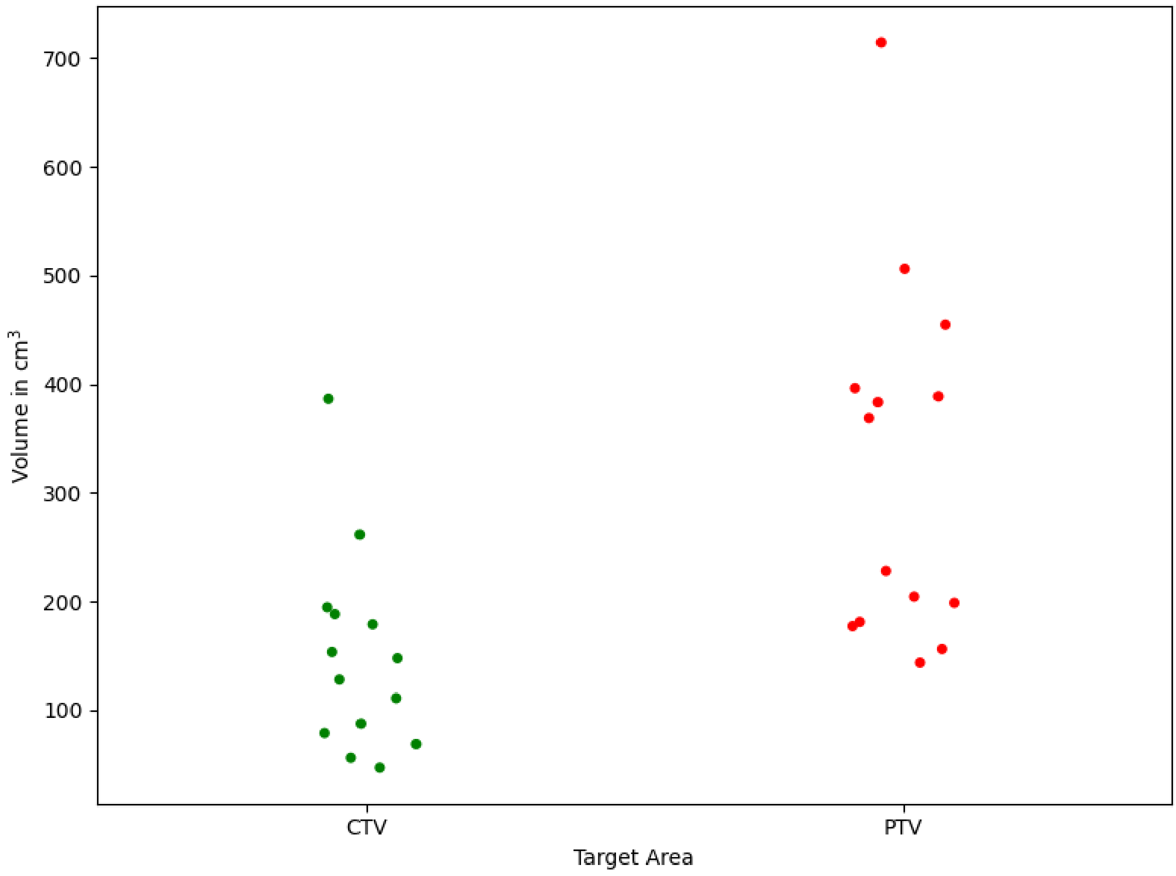

Figure S2 Scatterplot summarizes target volumes across all patients (n=15).

1.1. Supplementary materials D

Control group

Data from the previously published FAST-METS [1] study were used as a reference for comparison.

Both the original FAST-METS and the current FAST-METS 2.0 studies applied the same inclusion criteria, namely patients referred for palliative radiotherapy for non-mobile metastases, treated using a single fraction of 8 Gy, and having diagnostic imaging no older than six weeks prior to treatment.

The original FAST-METS cohort comprised 100 patients, whereas the current study included 15 patients. The median ages were 68 and 70 years, respectively. Both cohorts included a similar distribution of treatment and primary tumor sites, and all patients had a Karnofsky Performance Status (KPS) greater than 50 prior to treatment.

These findings indicate that both study populations were demographically and clinically comparable, thereby allowing for a valid comparison of workflow and treatment outcomes.

The treatment times for both patient cohorts were also comparable, with no significant differences observed (Wilcoxon rank-sum test, p=0.66).

[1] Nelissen, K.J., W. Verbakel, B.J. Slotman, J. Visser, and E. Versteijne, *Routine Use of Single Visit Online Adaptive Cone Beam Computed Tomography Guided Sim-Free Palliative Radiation Therapy.* Int J Radiat Oncol Biol Phys, 2025. 10.1016/j.ijrobp.2025.03.040.
